# Preparation of Hydrophilic Polysilsesquioxanes and Their Applications as Water Separation and Antifogging Coating Materials

**DOI:** 10.1002/tcr.70218

**Published:** 2026-07-23

**Authors:** Takashi Hamada, Yohei Adachi, Joji Ohshita

**Affiliations:** ^1^ Collaborative Research Laboratory Graduate School of Advanced Science and Engineering Hiroshima University Higashi‐Hiroshima Japan; ^2^ Department of Advanced Functional Materials Research Takasaki Institute for Advanced Quantum Science National Institutes for Quantum Science and Technology (QST) Takasaki Japan; ^3^ Smart Innovation Program Graduate School of Advanced Science and Engineering Hiroshima University Higashi‐Hiroshima Japan; ^4^ Division of Materials Model‐Based Research Digital Monozukuri (Manufacturing) Education and Research Center Hiroshima University Higashi‐Hiroshima Japan

**Keywords:** antifogging, coating material, polysilsesquioxane, reverse osmosis, separation membrane

## Abstract

Polysilsesquioxanes (PSQs) have been extensively studied as typical organic–inorganic hybrid materials that exhibit functionality and processability owing to organic substituents on silicon atoms, while also thermal and mechanical stability attributable to the inorganic siloxane (Si–O–Si) network structure. We describe herein the introduction of hydrophilic substituents into random PSQs to improve properties, such as water permeability and absorption capability, for developing reverse osmosis (RO) membranes for water separation and antifogging coating materials. Previously studied PSQ‐based RO membranes generally exhibited low water permeability, although they showed excellent heat and chlorine resistance. Recently, we introduced hydroxy‐containing substituents onto the silicon atoms of PSQs, enhancing the water permeability of PSQ‐based RO membranes. Scratch‐resistant antifogging coating materials are obtained by introducing amino, ammonium, and hydroxy units onto PSQ silicon atoms. The low durability of the initially studied materials was improved through chemical modification of the PSQ structures.

## Graphical abstract

Hydrophilic substituents were introduced onto the silicon atoms of random polysilsesquioxanes (PSQs) to improve water permeability and absorptivity for exploring the applications of PSQ materials as heat‐ and chlorine‐resistant water desalination reverse osmosis (RO) membranes and scratch‐resistant antifogging coating materials.

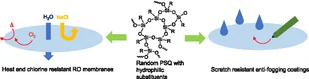

## 1 Introduction

Polysilsesquioxanes (PSQs) are siloxane‐based network polymers that can be efficiently synthesized through the hydrolysis/condensation of trifunctional silicon precursors RSiX_3_ (X = halogen, alkoxy, amino, etc.), as shown in Figure 1. PSQs have been extensively studied as typical organic–inorganic hybrid materials that exhibit functionality and processability owing to organic substituents on silicon atoms, while also thermal and mechanical stability attributable to the inorganic siloxane (Si–O–Si) network structure [1, 2, 3, 4, 5, 6]. From the general formula (RSiO_1.5_)_*n*_, which lies between those of linear polysiloxanes (R_2_SiO)_*n*_ and silica (SiO_2_)_*n*_, PSQs have fewer organic substituents than linear polysiloxanes and are therefore less organic. The organic substituents improve the processability of PSQs, enabling smooth preparation of coating films. For example, the sol–gel process, which involves sol formation through the oligomerization of precursors with water in diluted solutions, followed by coating the resultant sols onto substrates and calcination, readily provides gel films. Alternatively, PSQ polymers with an appropriate molecular weight can be coated onto a substrate with or without solvent and then calcined. The obtained PSQ films are studied for applications in various materials, including adhesives, heat insulators, encapsulants, and surface modifiers, owing to their excellent heat resistance and mechanical stability.

**FIGURE 1:**
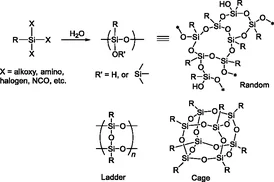
Synthesis of PSQs and their typical structures.

PSQs are categorized into three types on the basis of their structural characteristics: random, ladder, and cage (Figure 1). We focus on our recent advances in the applications of readily accessible random PSQs with hydrophilic substituents. Generally, PSQ silicon atoms bear simple organic substituents, and PSQs are used as hydrophobic materials. For example, it was demonstrated that the combination of hydrophobic cage [7, 8, 9, 10, 11, 12] and ladder [13, 14] PSQs with hydrophilic organic chains led to the development of amphiphilic polymers. Applications of some cage‐ and ladder‐type PSQs with hydrophilic substituents to amphiphilic materials have been also reported [15, 16, 17, 18, 19, 20]. Hydrophilic surface modifiers have been developed based on cage PSQs [21, 22]. Ladder and cage compounds containing ammonium or other ionic units have been reported as ionic liquids [23, 24, 25]. PSQs with sulfonate and phosphonate substituents have also been investigated as proton‐conducting materials [26, 27]. Additionally, amino‐functionalized PSQs, including random ones, have been studied as CO_2_ separation membranes [28]. However, less is known about the applications of PSQs as hydrophilic materials compared to hydrophobic PSQs. We describe herein the development of hydrophilic PSQ materials actively utilizing interactions with water. The introduction of hydrophilic substituents into random PSQs effectively improved water permeability and absorption capability, thereby enabling the development of robust water separation reverse osmosis (RO) membranes with heat and chlorine resistance, and antifogging coating materials with mechanical strength.

## 2 PSQs for Water Separation Membranes

### 2.1 Background

Water shortage is a significant global concern. Seawater desalination by membrane separation is expected to provide a straightforward solution as it features low energy consumption and a simple process compared with conventional distillation [29]. RO membranes based on organic polymers, such as aromatic polyamide, are widely used for water desalination as they exhibit high water permeability and salt rejection [30, 31, 32, 33]. However, organic RO membranes generally exhibit low thermal and chemical stability, limiting their use. To develop robust RO membranes, the modification of membrane structures, for example, composite formation of aromatic polyamide membranes with graphene oxide and carbon nanotubes, has been examined [34, 35, 36, 37, 38, 39, 40, 41, 42, 43, 44]. In 2011, Tsuru, Kanezashi, and coworkers reported the preparation of a PSQ‐based RO membrane from bis(triethoxysilyl)ethane (**BTESE**) for water desalination. Silica membranes prepared by hydrolysis/condensation of TEOS (tetraethoxysilane) are typically too dense to allow sufficient water permeability. In contrast, in the **BTESE**‐derived membrane, the ethylene linkages expand the silica network, making the membrane less dense, thereby enhancing water permeability (spacer method). Additionally, the **BTESE**‐derived membrane is stable—it is not degraded even after exposure to chlorine (sodium hypochlorite aqueous solution) for 35,000 ppmh and operation at 90 °C for over 200 h [45]. However, the **BTESE**‐derived membrane exhibits much lower water permeability than commercially available organic membranes, although NaCl rejection is as high as 98%. Hybridization of **BTESE** with polyamide to improve the stability has been studied by Zheng et al. [46].

Following this original finding by Tsuru, Kanezashi, and coworkers, we developed bis(triethoxysilyl)ethene (**BTESE2**) and bis(triethoxysilyl)ethyne (**BTESE3**) as new PSQ precursors in collaboration with the group of Tsuru and Kanezashi (Figure 2) [47]. Interestingly, the water permeability of **BTESE2**‐ and **BTESE3**‐derived membranes was improved by two‐ to tenfold with no significant decrease in NaCl rejection. It is likely that sterically more rigid ethenylene and ethynylene structures further expand the PSQ siloxane network, improving water permeability. Less‐hindered ethenylene and ethynylene structures are also responsible for the improved water permeability. Additionally, the higher polarity of unsaturated ethenylene and ethynylene units in **BTESE2** and **BTESE3** than the ethylene unit in **BTESE**, likely enhances the water affinity of PSQ membranes, thus increasing water permeability. Inspired by the idea that PSQ membrane performance could be readily tuned by precursor molecular design, we examined a variety of PSQ precursors for the preparation of water desalination RO membranes (Figure 2) [48]. Although we improved the water permeability of PSQ‐based membranes to some extent by increasing the rigidity and/or polarity of the PSQ precursors, the membranes still exhibited lower water permeability than commercial polyamide membranes. NaCl rejection and water permeability, two important parameters of water desalination RO membranes, are essentially in a trade‐off relationship, so it is generally difficult to improve both simultaneously. We needed new ideas to increase water permeability while maintaining NaCl rejection at a sufficiently high level.

**FIGURE 2:**
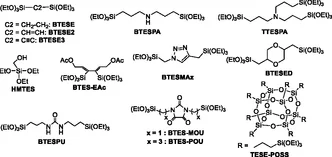
Examples of precursors of PSQ‐based RO membranes.

### 2.2 Fabrication of Membranes and Their Evaluation

The PSQ‐based RO membranes discussed herein were prepared using either an inorganic porous material or an organic nanofiltration membrane as the support membrane. On the support, PSQ sol prepared by hydrolysis/condensation of the corresponding precursor was coated and calcined to promote gelation, producing a PSQ gel layer (Figure 3) [49]. In this process, the PSQ sol particles should be larger than the pore size of the support membrane (1–2 nm) so that the sol does not penetrate the pores. However, calcination of sols with excessively large particle sizes may lead to the formation of grain boundary pores, making it difficult to fabricate a uniform PSQ membrane free of defects, such as cracks and pinholes. Particle size can be controlled primarily by adjusting the reaction time; however, a large amount of water should be used if the sol formation proceeds too slowly to complete the formation of sol particles with suitable sizes within a reasonable reaction period. In some cases, the reaction temperature, solvent, and precursor concentration are also adjusted to control the sol formation. Sol particle sizes were estimated by dynamic light scattering, and the reactions were continued until the sol particle sizes reached 2–10 nm. In addition to the appropriate sol particle size control, the calcination temperature should also be carefully determined. A high calcination temperature facilitates gelation, producing a dense membrane with high water separation selectivity. However, if the temperature is too high, thermal decomposition of organic groups occurs. Typically, organic support membranes that are thermally stable up to 150 °C are used to fabricate flexible PSQ‐based RO membranes, unless calcination at higher temperatures is required. Interfacial polymerization can also be used to prepare membranes with amino units on an organic support [50, 51, 52].

**FIGURE 3:**
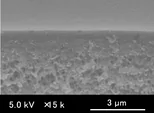
Cross‐sectional SEM image of a PSQ‐based RO membrane, where a PSQ layer is formed on top of an organic support, showing a smooth surface without defects. Reprinted from reference [49] with permission from the Chemical Society of Japan.

The resultant membrane was placed in a device containing 2,000 ppm NaCl aqueous solution, and water permeation was examined by providing a feed pressure of 1.5 MPa at room temperature. Generally, in the early stages of the water separation experiments, water permeability and NaCl rejection increase gradually, likely because the permeation of NaCl aqueous solution into the membrane increases membrane wettability and electrostatic repulsion between the salts absorbed in the membrane and those dissolved in the aqueous solution. The permeated aqueous solution was analyzed to determine water permeability and NaCl rejection after steady state was reached.

### 2.3 Hydroxy‐Containing PSQ Membranes

In order to increase the water affinity of PSQ membranes, we prepared PSQ membranes using hydroxymethyltriethoxysilane (**HMTES**, Figure 2) as a precursor [53, 54]. The resulting membranes showed enhanced, but still insufficient, water permeability. Recently, we examined the copolymerization of triethoxy‐3‐(glycidylpropyl)silane (**TEGPS**) and bis(triethoxysilylpropyl)amine (**BTESPA**), expecting that the ring‐opening nucleophilic addition of the amino group in **BTESPA** to the epoxy unit in **TEGPS** would generate a hydroxy bond, competing with the polymerization (Figure 4) [55]. As expected, the solid‐state NMR spectra indicated the formation of a ring‐opened structure in the resultant PSQ, and the **TEGPS–** **BTESPA** copolymer membranes exhibited enhanced water permeation compared with other PSQ‐based RO membranes prepared until then while maintaining good NaCl rejection. Membrane performance was dependent on the precursor ratio; the membrane prepared with an optimized **TEGPS**/**BTESPA** weight ratio of 1:2 showed approximately ninefold higher water permeance with slightly higher NaCl rejection than the **BTESPA** homopolymer membrane, and these data were comparable to those of commercially available aromatic polyamide membrane SW30 HR (Table 1) [60]. The membrane also exhibited high chlorine resistance. However, the membrane was stable only up to 60 °C; it rapidly deteriorated when water separation was examined at 80 °C. To improve thermal stability, we employed a glycidyl‐containing precursor with a polyhedral octasilsesquioxane (POSS) core, which was expected to enhance heat resistance owing to its high structural rigidity (**GPOSS** in Figure 5) and examined its copolymerization with **BTESPA** [56]. Although water permeability was slightly lower than that of the **TEGPS–** **BTESPA** membrane, heat resistance was significantly improved, and the **GPOSS** **–BTESPA** 1:5 membrane functioned as a heat‐resistant RO membrane for water separation, operative even at 100 °C. The membrane also exhibited high chlorine resistance. Similar studies utilizing a POSS precursor bearing carboxylic acid units on the corner silicon atoms (**POSS** **–COOH**) were reported by Yamamoto, Gunji, and coworkers [61, 62].

**FIGURE 4:**
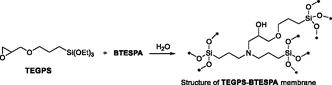
Copolymerization of **BTESPA** and **TEGPS**, producing a hydroxy unit‐containing PSQ‐based RO membrane.

**FIGURE 5:**
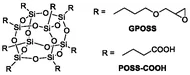
Structures of **GPOSS** and **POSS–COOH**.

**TABLE 1:** Performance of PSQ‐based RO membranes.

Precursor (weight ratio)	Water permeability, m^3^/m^2^sPa	NaCl rejection, %	Ref.
**BTESPA**	1.1 × 10^−13^	96	[49]
**TEGPS** **–BTESPA** (1/2)	9.6 × 10^−13^	97.5	[55]
**GPOSS** **–BTESPA** (1/5)	5.5 × 10^−13^	98.9	[56]
**HETESPU** **–BTESPA** (1/3)	1.9 × 10^−12^	95.9	[57]
**DHETESPU** **–BTESPA** (1/3)	1.9 × 10^−12^	96.8	[57]
**BHETESPU** **–BTESPA** (1/4)	1.0 × 10^−12^	98.2	[58]
**BTESEgU** **–BTESPA** (1/3)	1.5 × 10^−12^	96.4	[58]
**TTESPUEA** **–BTESPA** (1/7)	1.1 × 10^−12^	96.2	[58]
**BTESPA**–silica (100/1)	1.1 × 10^−12^	98.2	[59]
SW30HR	1.1 × 10^−12^	98.5	[60]

We also investigated urea‐containing precursors [57, 58]. Urea is a hydrophilic group that can form strong interunit hydrogen bonds [63]. First, *N*‐hydroxyethyl‐*N’*‐(triethoxysilylpropyl)urea (**HETESPU**) was prepared as a new PSQ precursor with a urea group by the reaction of ethanolamine and (triethoxysilylpropyl)isocyanate (Figure 6a) and its copolymerization with **BTESPA** was investigated [57]. It was anticipated that the hydroxyethylurea units would aggregate, forming a hydrophilic channel that facilitates water permeation through the **HETESPU**‐based PSQ membrane (Figure 6b). Water separation performance was dependent on the precursor ratio; as the **HETESPU** incorporation ratio increased, pinholes formed on the membrane surface significantly reduced NaCl rejection. However, at an optimized **HETESPU**:**BTESPA** ratio of 1:3, the membrane exhibited high water permeability (Table 1) and moderately high NaCl rejection. Atomic force microscope (AFM) analysis of the membrane surface suggested aggregate formation, as anticipated (Figure 6c). To further improve performance, we prepared a membrane using a new precursor, **DHETESPU**, as shown in Figure 7 and Table 1. The trimethylsiloxy groups were likely hydrolyzed during the sol–gel process, forming hydroxy groups. However, these **HETESPU**‐ and **DHETESPU**‐derived RO membranes exhibited only moderate thermal stability of up to 60 °C and slightly low chlorine resistance.

**FIGURE 6:**
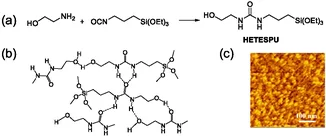
(a) Synthesis of **HETESPU**. (b) Illustration of possible aggregation in the PSQ membrane through hydrogen bonding. (c) AFM image of the **HETESPU–BTESPA** copolymer membrane surface. Reprinted with permission from reference [57]. Copyright 2022 American Chemical Society.

**FIGURE 7:**
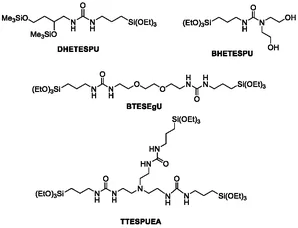
Structures of urea‐containing PSQ precursors.

To improve the stability of urea‐containing RO membranes, we designed three new precursors, **BHETESPU**, **BTESEgU**, and **TTESPUEA**, as shown in Figure 7. It is known that degradation of urea by chlorine is initiated by the formation of an N–Cl. Therefore, **BHETESPU** was designed to have only one N–H bond. **BTESEgU** and **TTESPUEA** have two and three triethoxysilyl units, respectively, which enhanced network formation in their membranes, thereby leading to higher stability [58]. Indeed, the copolymerization of **BHETESPU**, **BTESEgU**, and **TTESPUEA** with **BTESPA** yielded membranes whose performance was comparable to the **HETESPU–BTESPA** membrane, and the **BHETESPU–BTESPA**, **BTESEgU–BTESPA**, and **TTESPUEA–BTESPA** membranes showed enhanced chlorine resistance, with only a 1%–3% decrease in NaCl rejection, even when exposed to chlorine for 10,000 ppmh. In contrast, under the same conditions, the **HETESPU–BTESPA** membrane exhibited deterioration, reducing NaCl rejection by approximately 20% relative to the pristine membrane.

### 2.4 PSQ–Silica Hybrid Membranes

To develop a simple strategy to improve the water separation performance of PSQ‐based membranes, we examined the hybridization of PSQ with silica nanoparticles [59]. When **BTESPA** was stirred with water in ethanol in the presence of a small amount of hydrophobic silica nanoparticles (10 nm), a **BTESPA**–silica hybrid sol was obtained. The obtained sol was coated onto an organic support membrane and calcined, yielding an RO membrane with highly improved water permeability and NaCl rejection compared with the **BTESPA** homopolymer membrane (Table 1). The scanning electron microscope (SEM) image in Figure 8 shows the formation of a defect‐free membrane with well‐dispersed silica nanoparticles on the organic support membrane. Although the mechanism underlying the performance improvement is not yet fully understood, the interface between the silica nanoparticles and PSQ appears to play an important role. It is assumed that small amounts of silanol groups on the hydrophobic silica nanoparticle surface form siloxane bonds with PSQ upon hybrid formation. In addition, the **BTESPA**–silica hybrid membrane exhibited excellent heat and chlorine resistance. Indeed, the membrane did not degrade even when it was repeatedly heated four times from room temperature to 95 °C. No separation performance changes were observed upon exposure of the membrane to chlorine for 10,000 ppmh. The method employing hybrids with nanoparticles does not necessitate complex precursors and offers a straightforward approach to enhancing membrane performance. Integrating this method with the optimization of precursor structures appears promising for achieving superior performance.

**FIGURE 8:**
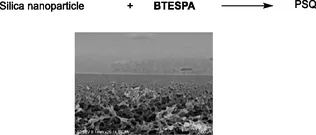
Cross‐sectional SEM image of BTESPA–silica nanoparticle hybrid film formed on an organic support membrane. Reproduced from reference [59]. Copyright (2022), with permission from Elsevier.

## 3 PSQs for Scratch‐Resistant Antifogging Coating Films

### 3.1 Background

Fogging is troublesome, making daily life uncomfortable or even dangerous at times. For example, fogging on automobile windshields reduces visibility, increasing the risk of accidents. Typically, defogger systems are installed in automobiles to clear fog from windshields by blowing dry warm air. However, this process is energy‐intensive. The development of robust antifogging coating materials that perform reliably across various conditions is highly anticipated. Fogging occurs when light is scattered owing to moisture condensing into water droplets on the surface of glass or other optical materials [64, 65, 66]. Examples of typical antifogging coating materials that prevent water droplet formation include hydrophilic [67, 68], super‐hydrophobic [69, 70, 71], and water‐absorptive coating materials [72, 73, 74]. Hydrophilic coatings, such as inorganic oxide and surfactant coatings, are used to spread condensed water effectively, forming a uniform thin water layer on the glass surface to avoid droplet formation. TiO_2_ is a typical coating material that exhibits excellent hydrophilicity. However, TiO_2_ coating is hydrophilic only under UV light, and the surfactant coating is not durable. Super‐hydrophobic water‐repellent coatings have also been studied in terms of nanoscale surface roughness, which is usually difficult to achieve, and the surfaces exhibit low scratch resistance. Although highly hydrophobic polymers, such as fluorocarbon‐containing polymers, can also be used as water‐repellent coating materials, they are usually not durable and are a concern for the environment. Water‐absorptive coating materials have attracted considerable attention as durable and effective antifogging coatings. Hydrophilic organic polymers are generally used as absorptive coating materials, but they exhibit poor scratch resistance, limiting their use. Hydrophilic polymers have been modified to improve scratch resistance. For example, hydrophilic organic polymers with a trialkoxysilyl unit at the polymer end were prepared to improve durability through chemical bonding to a glass surface [75, 76]. In general, however, water absorption and surface hardness are in a trade‐off relationship, making it challenging to enhance both simultaneously. PSQs have attracted attention because of their inorganic siloxane‐based network structures, which lead to high scratch resistance. Kaneko and coworkers recently demonstrated that well‐designed polymers with caged PSQ units linked by hydrophilic organic units exhibited high scratch resistance and antifogging properties [77, 78]. In the following subsection, we describe our efforts to develop easily accessible, random PSQ‐based, scratch‐resistant, antifogging coating materials.

### 3.2 Preparation of PSQ‐Based Antifogging Coating Films

We investigated several PSQs with hydrophilic groups on silicon atoms of random PSQs and developed antifogging coating materials with high scratch resistance (Figure 9) [79, 80, 81, 82, 83, 84, 85]. First, PSQ was synthesized as a viscous colorless transparent liquid from the corresponding trimethoxysilane or triethoxysilane with a hydrophilic substituent. The obtained PSQ was dissolved in an appropriate solvent, such as ethanol, to make an approximately 20 wt% solution. The PSQ solution was coated on a glass plate using an applicator, air‐dried, and calcined in air at approximately 180 °C to obtain a gel film on the glass substrate. To form films of hydroxy group‐containing **PSQ‐OH** and **PSQ‐Eg‐OH** [80, 81], and ammonium group‐containing **PSQ‐NH2+** and **PSQ‐SiMe‐NH2+** [82], PSQ gel films containing epoxy groups (**PSQ‐Ep** and **PSQ‐Eg‐Ep**) and amino groups (**PSQ‐NH2** and **PSQ‐SiMe‐NH2**) were produced, as described above. The films were immersed in an acidic aqueous or methanolic solution to convert them into diol and ammonium groups, respectively (Figure 10).

**FIGURE 9:**
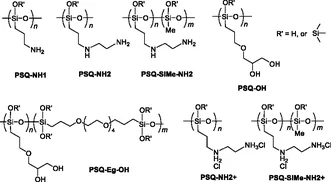
Chemical structures of PSQs for scratch‐resistant antifogging coating materials.

**FIGURE 10:**
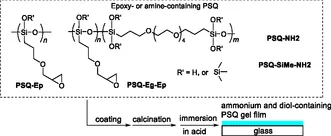
Preparation of coated glass plates with ammonium and diol units.

Table 2 lists the water uptake measured as an index of antifogging property and the scratch resistance or pencil hardness. The scratch resistance was determined by nanoindentation scratch tests of the obtained PSQ films. The data for a polyvinyl alcohol (PVA) film are also included for comparison. As shown in Figure 11, nanoindentation scratch tests were performed in three steps: (1) monitoring the original surface profile using a probe; (2) scratching the surface by loading weight on the probe, which increases proportionally to the distance; and (3) monitoring the postscratch surface profile. For the **PSQ‐NH2** film, the postscratch surface profile revealed almost complete recovery of the original surface, indicating that the scratch was primarily due to elastic deformation (Figure 11a). In contrast, the PVA film showed a large plastic deformation (Figure 11b). Water uptake and scratch resistance of PSQ films are typically in a trade‐off relationship, and the film properties are dependent on calcination temperature and time. Calcination at high temperatures and for extended periods generally reduces water uptake and increases scratch resistance. The immersion time in acidic solution to induce the chemical conversion of epoxy‐ and amino‐containing PSQ films into the corresponding hydroxy‐ and ammonium‐containing films also affected film performance. Therefore, well‐balanced data were selected from the data obtained under different conditions in Table 2 for each PSQ film. Compared with PVA film, PSQ‐based antifogging coating films exhibited comparable or slightly low water uptake and excellent scratch resistance. The films exhibited efficient antifogging properties, as illustrated in Figure 12 for **PSQ‐Eg‐OH**.

**FIGURE 11:**
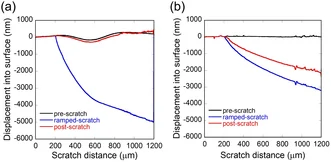
Nanoindentation scratch tests of (a) **PSQ‐NH2** and (b) PVA‐coated films (prescratch: original surface profile; ramped‐scratch: scratch profile obtained by loading weight on the probe, which increases proportionally to the distance; postscratch: postscratch surface profile). In the case of **PSQ‐NH2**, the postscratch surface profile indicated almost complete recovery of the original surface. Reprinted with permission from reference [79]. Copyright 2022 American Chemical Society.

**FIGURE 12:**
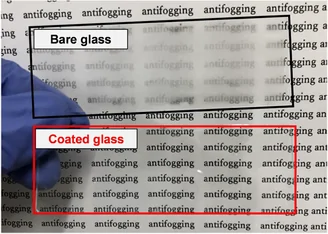
Antifogging test of **PSQ‐Eg‐OH**. The coated glass plate showed no fogging when exposed to water vapor. In contrast, the bare glass plate showed fogging under the same conditions. Reprinted with permission from reference [81]. Copyright 2022 American Chemical Society.

**TABLE 2:** Evaluation of PSQ‐based antifogging coating materials.

PSQ (*n*:*m*)	Water uptake, %	Scratch resistance or pencil hardness	Coloring	Durability	Ref.
**PSQ‐NH1**	34	—a	−	−	[79]
**PSQ‐NH2**	22	3.2	+	−	[79]
**PSQ‐OH**	19	2.7	−	−	[80]
**PSQ‐Eg‐OH** (1:1)	19	3.5	−	+	[81]
**PSQ‐NH2+**	29	4.3	−	−	[82]
**PSQ‐SiMe‐NH2+** (2:1)	17	3.7	−	+	[82]
**PSQ‐Eg‐NH2+** (1:1)	30	6H	−	+	[83]
**PSQ‐SiMe‐BnN** (1:4)	25	HB	−	+	[84]
**PSQ‐GlcT**_**1**_ **D**_**0**_	29	6H	−	+	[85]
**PSQ‐GlcT**_**1**_ **D**_**1**_	34	4H	−	+	[85]
PVA	27	0.5			[79]

a Not measured because of low surface smoothness.

Among these films, the **PSQ‐NH1** film was cloudy and exhibited high surface roughness, and its scratch resistance could not be evaluated. In contrast, **PSQ‐NH2** afforded uniform films with low surface roughness. However, the **PSQ‐NH2** films turned slightly yellow upon calcination in air, likely due to oxidation of the amino units of the films. To avoid discoloration, ammonium and hydroxy units were introduced as hydrophilic units to replace the amino units. As expected, the resultant ammonium‐ and hydroxy‐containing **PSQ‐NH2+** and **PSQ‐OH** films showed no discoloration when calcined in air. However, they exhibited low durability. When the **PSQ‐NH2+** and **PSQ‐OH** films were exposed to humid air, surface roughness increased with exposure time, likely due to film shrinkage caused by condensation of silanol units remaining in the films (see Figure 13a for **PSQ‐OH**). Condensation also affected water uptake. For example, as shown in Figure 14a, repeated exposure to dry and wet atmospheres gradually decreased water uptake of the **PSQ‐OH** film. To avoid shrinkage, spacer units were introduced, expecting that they would sterically separate the silanol units from each other, suppressing their condensation. The flexible spacers were also expected to absorb stress caused by condensation, if it occurred. As expected, **PSQ‐SiMe‐NH2+** and **PSQ‐Eg‐OH** films were highly durable, and no changes in surface roughness or water uptake were observed under the same conditions (see Figures 13b and 14b for **PSQ‐Eg‐OH**).

**FIGURE 13:**
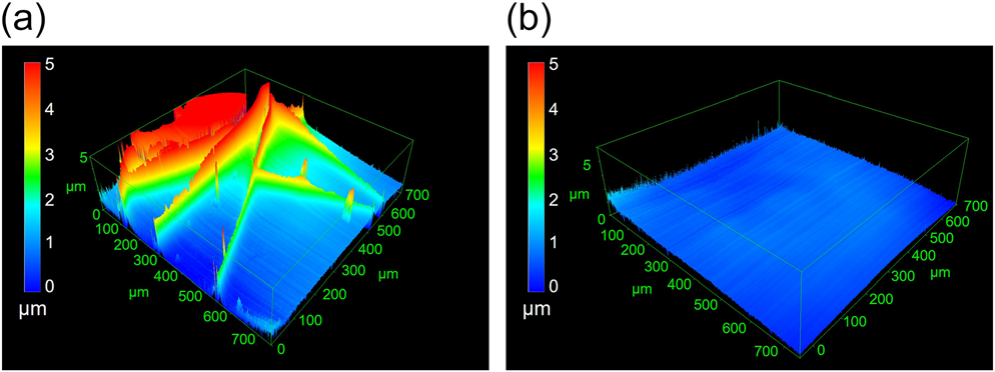
Laser microscope images of (a) **PSQ‐OH** and (b) **PSQ‐Eg‐OH** films after exposure to 95% relative humidity (RH) at 50 °C. Reprinted with permission from reference [81]. Copyright 2022 American Chemical Society.

**FIGURE 14:**
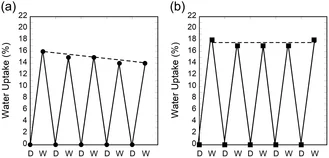
Results of dynamic vapor sorption experiments for (a) **PSQ‐OH** and (b) **PSQ‐Eg‐OH** films, showing water uptake changes of the films during repeated exposure to RH of 20% (D) and 95% (W) for 2 h at 30 °C. Reprinted with permission from reference [81]. Copyright 2022 American Chemical Society.

As described above, **PSQ‐SiMe‐NH2+** and **PSQ‐Eg‐OH** films exhibited high durability. However, their water uptake values were 17% and 19%, respectively, which were lower than those of other films and might have suppressed their antifogging performance. To improve the water uptake of PSQ films while retaining high scratch resistance and durability, we introduced both ammonium and tetraethylene glycol units into the PSQ structures (**PSQ‐Eg‐NH2+**) (Figure 15) [83]. As expected, the ammonium and tetraethylene glycol units exerted synergetic effects, furnishing coating materials with the desired properties, high durability and high water uptake.

**FIGURE 15:**
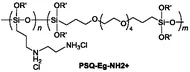
Structure of **PSQ‐Eg‐NH2+**.

Although the **PSQ‐Eg‐NH2+** film exhibited excellent antifogging properties, scratch resistance, and durability, the preparation required treating precursor films with a harsh reagent, HCl in methanol. To circumvent this, we prepared an ammonium‐containing PSQ film using a process that required no acid treatment [84]. PSQ with benzyl chloride units (**PSQ‐SiMe‐BnN**) was prepared through the copolymerization of benzyl chloride‐containing precursor and triethoxymethylsilane. Then, **PSQ‐SiMe‐BnN** was coated on a glass substrate, calcined at 180 °C for 3 h, and immersed in a THF solution of trimethylamine to convert the benzyl chloride groups into ammonium chloride units (**PSQ‐SiMe‐BnNMe+**), as shown in Figure 16. The film exhibited moderate surface hardness (pencil hardness index: HB), excellent antifogging property with 25% water uptake, and high transparency.

**FIGURE 16:**
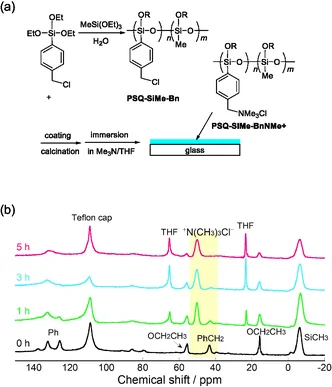
(a) Preparation of **PSQ‐SiMe‐BnNMe+** film and (b) solid‐state ^13^C NMR spectra of **PSQ‐SiMe‐Bn** film before and after immersion in 13 wt% THF solution of Me_3_N at room temperature for 1, 3, and 5 h. Reproduced from reference [84] with permission from the Royal Society of Chemistry.

To avoid post‐treatment, PSQ coating films functionalized with highly hydrophilic glucose units were prepared [85]. Tri‐ and diethoxysilane (**GlcTES** and **GlcDES**) monomers with glucose units were polymerized via a sol–gel process to form highly transparent coating films (Figure 17 and Table 2). The films exhibited excellent antifogging properties with high water uptakes, attributed to the abundance of hydroxyl groups on the glucose units. It was also noted that the films exhibited high durability and mechanical stability.

**FIGURE 17:**
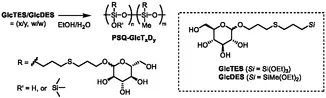
Preparation of glucose‐containing PSQ.

## 4 Summary and Outlook

Balancing material functionality and stability often involves trade‐offs, making it challenging to enhance both simultaneously. However, the structural features of PSQs as organic–inorganic hybrid materials, whose properties reflect the advantages of both organic and inorganic units, enabled the development of robust materials with high functionality. Hydrophilic substituents were introduced onto the silicon atoms of random PSQs to improve water permeability and absorptivity for exploring the applications of PSQ materials as water desalination RO membranes and antifogging coating materials. During the development of PSQ materials, we carefully designed the chemical structures of the organic substituents to concurrently optimize the trade‐off between water permeability and salt rejection, water separation properties and stability, and antifogging ability and scratch resistance, or to enhance one property while maintaining the other. We successfully enhanced the performance incrementally to achieve the desired functionalities. Finally, highly robust RO membranes with high performance comparable to commercial polyamide‐based membranes and effective antifogging coating materials with excellent scratch resistance and durability were developed. This study offers significant insights into the molecular design strategies for regulating the properties and functionalities of PSQ. Further studies on new PSQ‐based materials are in progress, and the results will be reported in due course.

## Author Contributions

T.H. and Y.A. contributed to the investigation of antifogging materials, the conceptualization of the study, and supervision of the research. Y.A. also supervised the experiments related to the RO membrane study. J.O. contributed to the conceptualization and project administration, supervised the overall project, and wrote the original draft of the manuscript. T.H., Y.A., and J.O. edited the manuscript.

## Funding

This work was supported by Japan Society for the Promotion of Science (Grant JP26K01558).

## Conflicts of Interest

The authors declare no conflicts of interest.

## Data Availability

The data that support the findings of this study are available from the corresponding author upon reasonable request.
